# Internal Consistency of Event-Related Potentials Associated with Cognitive Control: N2/P3 and ERN/Pe

**DOI:** 10.1371/journal.pone.0102672

**Published:** 2014-07-17

**Authors:** Wim J. R. Rietdijk, Ingmar H. A. Franken, A. Roy Thurik

**Affiliations:** 1 Department of Applied Economics, Erasmus School of Economics, Erasmus University Rotterdam, Rotterdam, The Netherlands; 2 Institute of Psychology, Erasmus University Rotterdam, Rotterdam, The Netherlands; 3 Panteia, Zoetermeer, The Netherlands; 4 GSCM-Montpellier Business School, Montpellier, France; University of Texas at Dallas, United States of America

## Abstract

Recent studies in psychophysiology show an increased attention for examining the reliability of Event-Related Potentials (ERPs), which are measures of cognitive control (e.g., Go/No-Go tasks). An important index of reliability is the internal consistency (e.g., Cronbach's alpha) of a measure. In this study, we examine the internal consistency of the N2 and P3 in a Go/No-Go task. Furthermore, we attempt to replicate the previously found internal consistency of the Error-Related Negativity (ERN) and Positive-Error (Pe) in an Eriksen Flanker task. Healthy participants performed a Go/No-Go task and an Eriksen Flanker task, whereby the amplitudes of the correct No-Go N2/P3, and error trials for ERN/Pe were the variables of interest. This study provides evidence that the N2 and P3 in a Go/No-Go task are internally consistent after 20 and 14 trials are included in the average, respectively. Moreover, the ERN and Pe become internally consistent after approximately 8 trials are included in the average. In addition guidelines and suggestions for future research are discussed.

## Introduction

Event-related potentials (ERPs) of cognitive control are increasingly used in clinical studies to examine the relevance in several forms of psychopathology [1], such as addiction [2] and obsessive-compulsive disorder [3]. Although ERPs have certain advantages over self-reporting (e.g., they are more objective) and behavioral measures (e.g., they provide more information on the neural level), relatively little attention has been paid to their psychometric properties, especially their reliability [4]. Reliability is a key psychometric criterion of physiological tasks [5],[6], and it is a necessary prerequisite to demonstrate their validity (i.e., the degree to which an ERP represents the intended underlying construct) [5],[6],[7],[8].

Reliability is frequently examined in terms of internal consistency (e.g., Cronbach's alpha) [7],[8]. The internal consistency of an ERP is defined as the similarity of the ERP across trials in a single task [8]. ERPs are usually derived by averaging (many) trials, and if the trial-to-trial waveforms are unreliable, the participant's average will also be unreliable (i.e., less internally consistent) [7],[8],[9]. Olvet and Hajcak [1] and Cohen and Polich [9] were among the first to examine the internal consistency of several cognitive control task ERPs, such as the ERN, Pe, and P300. Among others, Riesel et al. [4] stated that there is ample room for more studies examining the reliability (especially, the internal consistency) of ERPs in cognitive control tasks (e.g., [1],[4],[8],[9],[10],[11]), such as the N2 and P3 in a Go/No-Go task. This study addresses the internal consistency of four frequently used ERP measures in two cognitive control tasks: the N2/P3 components measured during a Go/No-Go task, and the ERN/Pe components measured during an Eriksen Flanker task.

In a Go/No-Go task, two major ERP components are enhanced for No-Go trials compared with Go trials, suggesting that they reflect brain activity related to inhibitory control. The first component is the N2, which is a negative wave emerging approximately 200–300 ms after stimulus onset. The N2 reflects the first stage of inhibition, and/or it is related to conflict monitoring [12],[13],[14]. The other ERP component is the P3, which is a positive wave emerging approximately 300–500 ms after stimulus onset. Several studies suggest that the P3 reflects a later stage of the inhibition process that is closely related to actual inhibition of the motor response in the premotor cortex [13]. Previous studies have reported differences in the electrophysiological correlates of inhibitory control (i.e., the N2 and P3) that are driven by variations of the specific characteristics of the Go/No-Go task set up (e.g., single, multiple and semantic Go/No-Go stimuli) [15]. Therefore, it is important to understand these variations and study the consequences for the internal consistency of the electrophysiological measures of inhibitory control (i.e., the N2 and P3) [15]. In a previous study, Clayson and Larson [14] examined the internal consistency of the N2 in an Eriksen Flanker task and found an internally consistent N2 after 30 trials. Furthermore, Cohen and Polich [9] found the P3 to be internally consistent after 21 trials, measured in an oddball task. To our knowledge, ours is the first study to examine the internal consistency of both the N2 and P3 in a Go/No-Go task.

Previous research also identified two major ERPs that are enhanced for incorrect behavioral response trials (i.e., an error) compared with correct behavioral response trials, the Error-Related Negativity (ERN) and Positive error related wave (Pe) [16],[17]. The ERN is an automatic response-locked negative deflection, emerging between 0–150 ms after the onset of an incorrect behavioral response [18],[19]. The second positive deflection is the Pe, which peaks around 200–400 ms after the onset of an erroneous behavioral response. Although there is discussion about the exact meaning of the Pe [20], most studies indicate that the Pe is related to error recognition [20],[21],[22],[23]. Olvet and Hajcak [1] and Pontifex et al. [11] found an internally consistent ERN and Pe after 6 and 8 trials were included to the participant's average, respectively.

In cognitive control tasks, the participants usually perform about 500 trials of a speeded reaction time task in relatively rapid succession. Errors and correct No-Go trials (i.e., successful inhibition of a participant's motor response) tend to be rare, resulting in a relatively low number of trials in the ERP averages. In fact, the number of trials for these conditions and participants varies greatly [1],[9],[10]. It has been suggested that only 6 and 8 trials are required for ERN and Pe, respectively [1]. However, guidance on the actual number of trials required to obtain an internally consistent ERP component for the N2 and P3 is largely lacking [1],[14]. As a result, the current study is set up to test the internal consistency of the N2 and P3 in a Go/No-Go task. Moreover, to ensure the quality of our inferences about the internal consistency of the N2 and P3, we attempt to replicate the results of previous studies that address the internal consistency of the ERN/Pe in the same sample [1],[11],[22],[23].

## Method

### Participants And Procedure

118 healthy right handed participants (*M_age_* = 21.7 years, *SD_age_* = 2.8, 61 males) participated in the electroencephalographic (EEG) task. Data from 10 participants were not analyzable due to computer errors during recording of the data. Only participants with at least 30 correct No-Go trials (*N* = 95, 87%) were included in the EEG analysis. Additionally, only participants with at least 14 errors in the Eriksen Flanker (*N* = 70, 65%) were included. These sample selection criteria, and sample inclusion rates are similar to that of Olvet and Hajcak [1], Pontifex et al. [11], and Meyer et al. [22],[23]. Using an online questionnaire, participants were screened for previous brain surgeries, pregnancy, or history of psychiatric disorders (no participants had to be excluded due to these criteria). Participants were asked not to drink coffee or smoke for 1.5 hours before the experiment. The study was conducted in accordance with the Declaration of Helsinki, and written consent was obtained from each participant prior to participation. The study was approved by the Ethics Committee of the Erasmus Medical Center, Erasmus University Rotterdam.

### Tasks

Participants performed a Go/No-Go task [24]. A letter (A, I, E, O, or U) was presented for 200 ms. Each stimulus was followed by a black screen for a randomly varying duration (1020 ms–1220 ms) [24],[25]. Participants were instructed to respond to the letters in the Go trials by pressing a button with the index finger as fast as possible, and in the No-Go trials, participants were instructed to withhold their response (i.e., when the letter was similar to the previous letter). The task had 500 trials, 125 of which were No-Go trials (25%) [25].

Participants also performed an Eriksen Flanker task (200 congruent trials: SSSSS, HHHHH; and, 200 incongruent trials: SSHSS, HHSHH) [26],[27]. Participants were instructed to respond to the central letter. On a response box, they had to press H with their right index finger when the central letter was an H and S with their left index finger if the central letter was an S. Each trial started with a fixation cue (∧) for 150 ms. Letter strings were presented for 52 ms, followed by a blank screen for 648 ms. The participants had 700 ms from stimulus onset to respond. At the end of the respond period, a feedback symbol appeared indicating whether the response was correct (ooo), incorrect (xxx), or too late (!). An interval of 100 ms was used [27].

### Erp Measurement & Statistical Analysis

EEG was recorded using a Biosemi Active-Two amplifier system (Amsterdam, the Netherlands) at 32 scalp sites (positioned following the 10–20 International System and two additional electrodes: FCz and CPz) with active Ag/AgCl electrodes mounted in an elastic cap. Six additional electrodes were attached to the left and right mastoids, the two outer canthi of both eyes (HEOG), and the infraorbital and supraorbital region of the right eye (VEOG). All signals were digitalized with a sample rate of 512 Hz and 24-bit A/D conversion, with a band pass of 0–134 Hz. The data were off-line referenced to compute mastoids. Off-line, EEG and EOG activities were filtered with a band pass of 0.15–30 Hz (phase shift free Butterworth filters; 24 dB/octave slope). During offline processing, no more than four bad channels per participant were removed from the EEG signal, and new values per channel were calculated using topographic interpolation [24]. Data were segmented in epochs of 1000 ms (−200–800 ms after stimulus presentation) and 700 ms (−100–600 ms after the response) for inhibitory control and error processing, respectively [24],[25],[27]. The average of 200 ms before stimulus onset in the Go/No-Go task and 100 ms before the response in the Eriksen Flanker period served as a baseline which was subtracted from all subsequent time points [25],[27]. Segments with incorrect responses (i.e., false alarm for No-Go trials, incorrect Go response, or false alarms for Eriksen Flanker trials) were all excluded from the EEG analysis [24],[25]. After ocular correction [28], epochs, including an EEG signal exceeding ±100 µV, were excluded from the average [23]. All epochs were also visually inspected for other artifacts. Average ERP waves were calculated after baseline correction for artifact-free trials at each scalp site in each condition.

Go/No-Go inhibitory control studies have predominantly examined and observed inhibition-related N2 and P3 effects at Fz, Cz, Pz (e.g., [29],[30],[31],[32]). Therefore, in the current study we examine the internal consistency of the N2 and P3 at Fz, Cz, and Pz. The N2 is defined as the average value in the 175–250 ms time interval after stimulus onset [24],[25]. The P3 is defined as the average value in the 300–500 ms time interval after stimulus onset [25]. In the Eriksen Flanker task, the ERN is defined are the as the average value of FCz in the 25–75 ms time segment after response onset. The Pe is defined as the average value of Pz in the 200–400 ms time segment after response onset [24],[25]. Note that later on in the study, the grand average waveform figures represent the difference waveforms (No-Go – Go and error – correct) of the electrodes important in the Go/No-Go (Fz, Cz, Pz) and Eriksen Flanker (FCz and Pz) task, respectively. The grand average difference waveforms are more informative for observing the temporality of the ERP measures, compared to the average waveforms of the Go and No-Go correct and Eriksen Flanker error and correct trials separately. However, in our analysis we took the amplitudes for correct No-Go N2 and P3 and ERN and Pe error trials as the variables of interest, similar to [1],[11],[22],[23]. The separate figures for Go/No-Go and error/correct trials are available upon request from the corresponding author.

The current study employed a methodology similar to that described by Olvet and Hajcak [1], Pontifex et al. [11], and Meyer et al. [22],[23]. For the ERPs of inhibitory control and error processing, we measured the average of N2/P3 and ERN/Pe trials, respectively. Random pairs of trials were included in the average (i.e., 2, 4, 6, 8, 10, …, and the participants' average, across all trials), and paired *t*-tests were used to determine statistically significant differences. Signal-to-Noise ratios (SNRs) were estimated using a process available in Brain Vision Analyzer Version 2.0 software (www.brainproducts.com). First, noise is estimated by summing the squares of the difference between each data point and the average EEG value; this sum is then divided by the number of data points minus one. Second, average total power is estimated by taking the average of the squared values of each data point. Average power of the signal then equals the average total power minus the average noise power [1]. SNRs of the trial pair averages were assessed using paired *t*-tests. Additionally, we assessed internal consistency measuring the correlation between these smaller trial averages and the N2/P3 and ERN/Pe participants' average (i.e., all trials), and Cronbach's alpha when an increasing number of trials were included in the average [1],[11],[22],[23], both available in SPSS 19.0. The thresholds in the current study are similar to previous studies, where internal consistency is indicated when correlations reached 0.8 and Cronbach's alpha reached 0.6 [1],[11],[22],[23].

## Results

### Inhibitory Control

**Figure 1:**
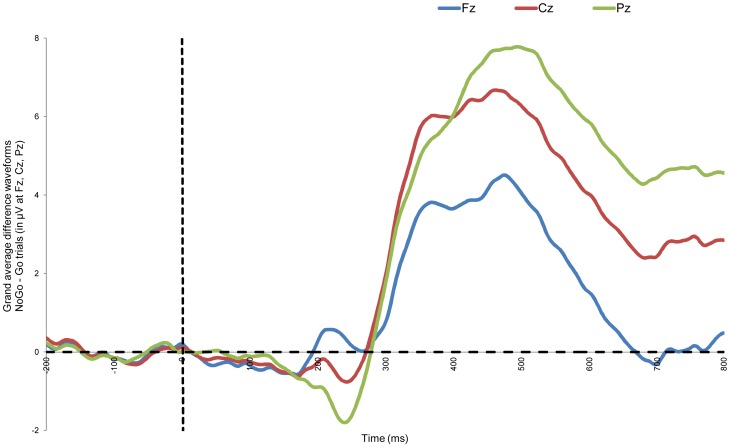
Grand average difference waveform: No-Go – Go trials. Figure 1 presents the grand average difference waveforms (i.e., average of all trials, across all participants) of the No-Go minus Go trials for electrodes Fz, Cz, and Pz. Note: we use the grand average difference waveforms for this figure as this is more informative compared to separate waveforms of No-Go correct trials and Go correct trials. However, in further analysis we took the amplitude for correct No-Go trials N2 and P3 at the midline electrodes Fz, Cz and Pz as the variables of interest.

**Figure 2:**
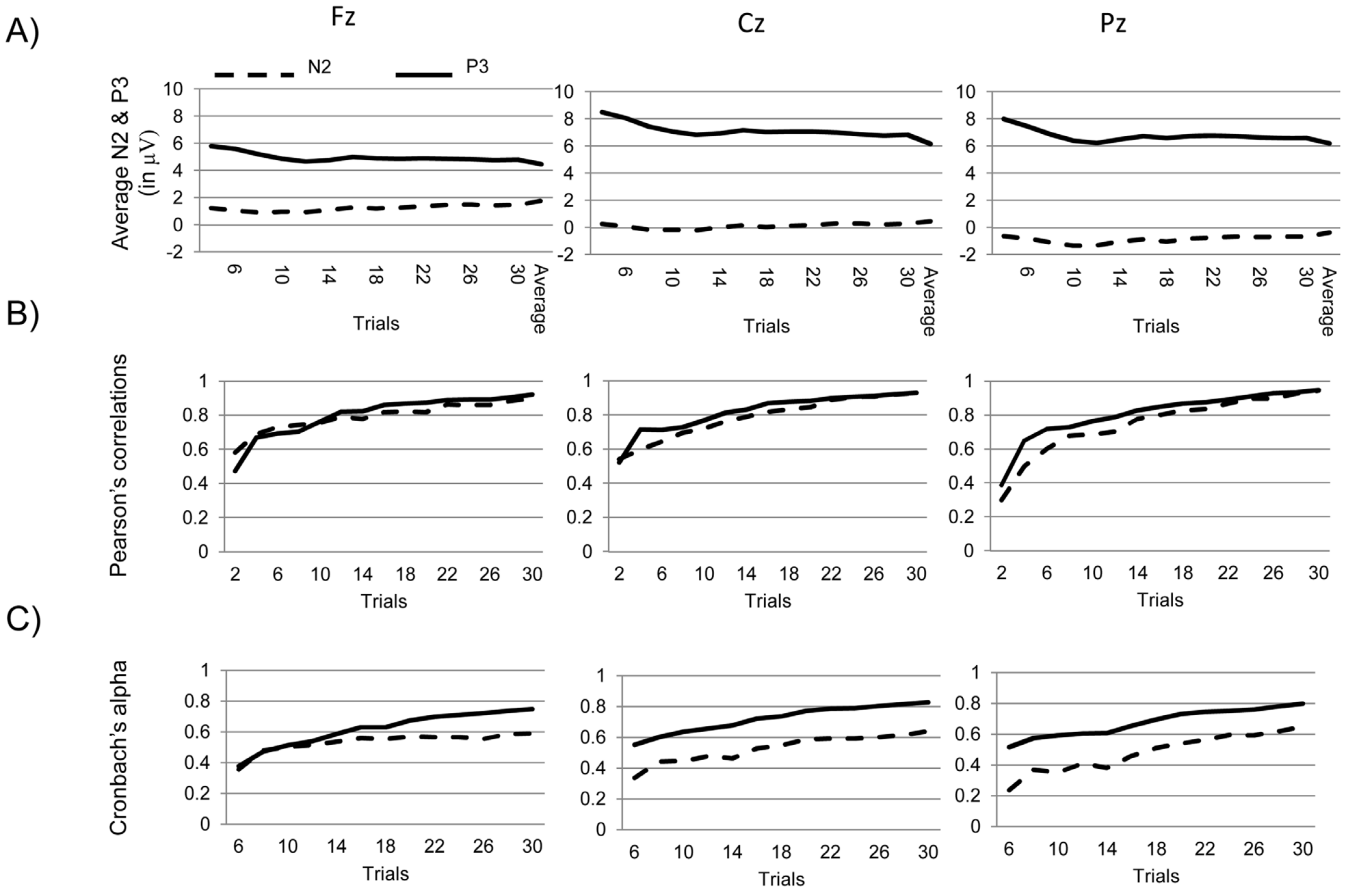
Correct No-Go N2 and P3– Internal consistency analysis. Figure 2 presents (A) the average N2 and P3, (B) Pearson's correlations, and (C) Cronbach's alpha as progressively more trials are included in the participants' average, all for the three midline electrodes Fz (left), Cz (middle), and Pz (right). The average presented in this figure refers to the grand average (all trials and all participants).

The purpose of this study is to examine internal consistency of the N2 and P3 in a Go/No-Go task. On average, the participants had 73.87 (SD = 19.87; 60% No-Go correct) correct No-Go trials (i.e., participants successfully inhibited their motor response while performing the task). Figure 1 presents the grand average difference waveforms for Go/No-Go task for the midline electrodes Fz, Cz, and Pz. Moreover, Figure 2 presents for all three midline electrodes the average (Figure 2A) and Pearson's correlations (Figure 2B), and the Cronbach's alpha (Figure 2C) all as a function of an increasing number of trials. Paired *t*-tests were performed using the N2 area measures, for all three midline electrodes (Fz, Cz, and Pz). Significant differences were only observed for electrodes Fz (30 vs. participants' average, *p*<0.05), and Pz (18 vs. 20 trials, and 30 vs. participants' average, *p*<0.05), while all other pairs comparing increasing numbers of trial averages (2 vs. 4 trials, 4 vs. 6 trials, 6 vs. 8 trials, 8 vs. 10 trials, 10 vs. 12 trials, …, 28 vs. 30 trials, and 30 trials vs. participants' average (i.e., all trials) were insignificant (all *p*s>0.05); this suggests that the N2 average is still relatively instable after 30 trials.

When comparing increasing trial numbers for the P3 significant differences at the three electrodes (Fz, Cz, Pz) were found for Fz (6 vs. 8 trials, *p* = 0.02; 8 vs. 10 trials, *p* = 0.04; 14 vs. 16 trials, *p* = 0.04), while all other pairs comparing increasing numbers of trial average were insignificant (all *p*s>0.05). Significant differences between increasing trials averages were found for Cz (6 vs. 8 trials, *p* = .018; 8 vs. 10 trials, *p* = .043; 14 vs. 16 trials, *p* = .045; 30 vs. grand average, *p* = .013), while all other pairs comparing increasing number of trial averages were insignificant (all *p*s>0.05). Significant differences between increasing trials averages were found for Pz (6 vs. 8 trials, *p* = .019; 26 vs. 28 trials, *p* = .039; 30 vs. grand average, *p* = .02), while all other pairs comparing increasing number of trial averages were insignificant (all *p*s>0.05). This suggests that the P3 is still relatively instable after 30 trials. Estimates of the SNR for N2 and P3 at Fz, Cz and Pz were also examined. SNR scores for the Fz electrode, starting with at least 6 errors, ranged from 0.43 to 0.14. Paired *t*-tests show that there were significant differences for 6 vs 8 trials, 8 vs. 10 trials, 10 vs. 12 trials, 22 vs. 24 trials, 24 vs. 26 trials, 28 vs. 30 trials and 30 vs. participants' average (*p*<0.05). SNR scores for the Cz electrode, starting with at least 6 errors, ranged from 0.67 to 0.28. Paired t-tests show that there were significant differences for 6 vs. 8 trials, 8 vs. 10 trials, 10 vs.12 trials, 16 vs. 18 trials, 22 vs. 24 trials, 24 vs. 26 trials, 30 vs. participants' average (*p*<0.05). SNR scores for the Pz electrode, starting with at least 6 errors, ranged from 0.61 to 0.30. Paired *t*-tests show that there were significant differences for 6 vs. 8 trials, 8 vs. 10 trials, 24 vs. 26 trials and 30 vs. participants' average (*p*<0.05). Taken together, one can conclude that the signal-to-noise ratio remains relatively unstable even when including as many as 30 trials.

Additionally, we explored the relationship between each trial average and the N2/P3 participants' averages using Pearson's correlation coefficient for Fz, Cz and Pz (Figure 2B). All pairs were highly significant (*p*<0.001), suggesting that individual trial averages share a degree of similarity with the participants' average when including only a couple of ERP trials. However, high correlations (*r*s>0.8; i.e., higher internal consistency) were reached after including 18 and 14 trials to the N2 and P3 averages, respectively. These data indicate that the ERP measures become similar to the participants' average (i.e., across all trials) after including 18 trials for both the N2 and P3.

Next, we determined the Cronbach's alpha for the N2 and P3 as progressively more trials were considered (Figure 2C). They both show an increasing trend. However, in order to obtain an adequate Cronbach's alpha (α>0.6) for the N2, at least 20 trials should be included in the participants' average. For the P3, an adequate Cronbach's alpha (α>0.6) was obtained after 14 trails were included in the average. It is important to note that the Cronbach's alpha for the N2 remains low compared to that for the P3. Taken together, these data demonstrate that in order to obtain an internally consistent estimate for the N2 and P3, 20 and 14 trials are required taking into account both the Pearson's correlations and Cronbach's alpha analyses, respectively.

### Error Processing

**Figure 3:**
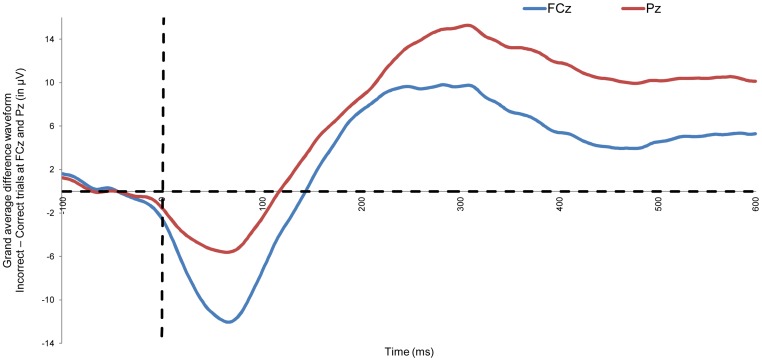
Grand average difference waveform: error - correct trials. Figure 3 presents the grand average difference waveforms (i.e., average of all trials, across all participants) of the error minus correct trials in the Eriksen Flanker task. Note: we use the grand average difference waveforms for this figure as this is more informative compared to separate waveforms of error and correct trials. However, in further analysis we took the amplitude for ERN (at FCz) and Pe (at Pz) error trials as the variables of interest.

**Figure 4:**
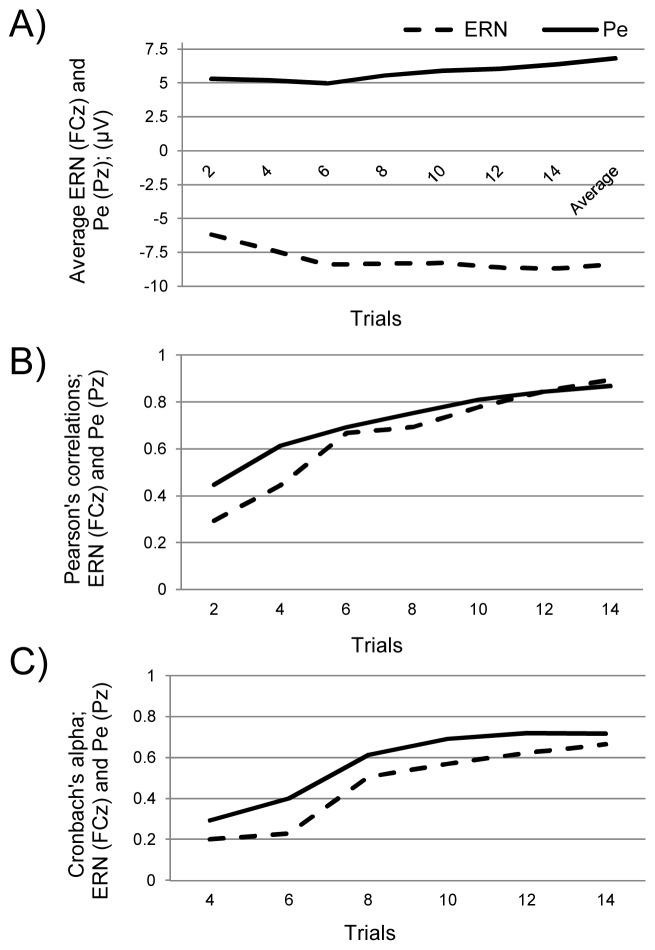
Error trials – Internal consistency analysis. Figure 4 presents the (A) average ERN and Pe, (B) Pearson's correlations, and (C) Cronbach's alpha as progressively more trials are included in the participants' average, for the ERN (at FCz) and Pe (at Pz). The average presented in this figure refers to the grand average (all trials and all participants).

To support the quality of our results regarding the internal consistency of the N2 and P3 in a Go/No-Go task, we attempted to replicate previous findings regarding the internal consistency of the ERN and Pe initially performed by Olvet and Hajcak [1]. On average, the participants made 26.31 errors (SD = 17.06) while performing the Eriksen Flanker task. The grand average difference waveforms for the Eriksen Flanker task for the electrodes FCz and Pz are presented in Figure 3. Moreover, Figure 4 presents for all three midline electrodes the average (Figure 4A), Pearson's correlation (Figure 4B), and the Cronbach's alpha (Figure 4C) as a function of an increasing number of trials. Paired *t*-tests were performed on the ERN area measures, and significant differences were observed only when comparing increasing numbers of trial averages for 4 vs. 6 trials (*p* = 0.03), and 6 vs. 8 trials (*p* = 0.03), while all other pairs were statistically insignificant (2 vs. 4 trials, 8 vs. 10 trials, 10 vs. 12 trials, 12 vs. 14 trials, and 14 vs. participants' average [i.e., all trials]; all *p*s>0.05); meaning that the average became stable after 8 trials were added to the participants' average. For the Pe, no significant differences were found (*p*>0.05); meanings that the Pe was relatively stable after 4 trials were included in the participants' average.

We also estimated the SNR for the ERN and Pe. SNR scores for the ERN starting with at least 6 errors ranged from 0.43 to 0.29, which is comparable to the magnitude reported in previous studies [1],[11]. For the ERN, only significant difference between SNR of trials averages 6 vs. 8 trials, 8 vs. 10 trials, and 10 vs. 12 trials, 12 vs. 14 trials (*p*<0.05), while for 14 trials vs. participants' average (*p*>0.05) was insignificant different. This means that after 14 trials the ERN signal-to-noise ratio became stable. As for the Pe SNR significant differences were observed for 12 vs. 14 trials and 14 trials vs. participants' average (*p*<0.05). This means that signal-to-noise for the Pe remained relatively unstable after 14 trials were included in the participants' average.

Additionally, we explored the relationship between each trial average and the ERN/Pe grand average using Pearson's correlation coefficient (Figure 4B). All pairs were highly significant (all *p*s<0.001), suggesting that individual trial averages share a degree of similarity with the participants' average when including only several ERP trials. However, the ERN and Pe trial averages showed high Pearson's correlations (i.e., higher internal consistency) after approximately 8 trials (*r*s>0.8) were included in the participants' average.

We also calculated the Cronbach's alpha for the ERN and Pe as progressively more trials were considered (Figure 4C). The Cronbach's alpha for the ERN and Pe were adequate (α>0.6) after 8 trials were included in the participants' average. Thus, the ERN and Pe were both internally consistent around 8 trials were included in the participants' average, respectively.

## Discussion

The present study examined the minimum number of trials required to obtain an internally consistent measure for ERPs in cognitive control tasks, the N2 and P3 in a Go/No-Go task and the ERN and Pe in an Eriksen Flanker task. The N2 in the Go/No-Go task displayed a less favorable internal consistency pattern compared to the Eriksen Flanker task ERPs. In the Go/No-Go task, the N2 showed high Pearson's correlation coefficients after 14 trials were included in the participants' average. However, adequate Cronbach's alpha was obtained only after approximately 20 trials. This suggests that approximately 20 trials are required to obtain an internally consistent estimate for the No-Go N2. As for the P3 in the Go/No-Go task, high Pearson's correlation coefficients were reached after 14 trials were included in the participants' average, and an adequate Cronbach's alpha was already obtained after including 8 trials. Thus, 14 trials are required to obtain an internally consistent estimate for the P3. Cohen and Polich [9] found an internally consistent P3 in an oddball task after 21 trials were included in the participants' average.

In addition, we replicate in the same sample the study by Olvet and Hajcak [1], Pontifex et al. [11], and Meyer et al. [22],[23]. In the current study, we found that approximately 8 trials are required to obtain an internally consistent estimate for the ERN and Pe. These recommendations are similar to previous studies [1],[11],[22],[23].

In the current design of the Go/No-Go task, participants are required to withhold a response when a letter (A, E, I, O, or U) was repeated. This adds two components to the Go/No-Go task: a working memory component and a response conflict component (i.e., in which a participant must withhold a response to a stimulus to which the participant just responded). Maguire et al. [15],[31] found that both the N2 and P3 amplitudes decrease with task difficulty (e.g., adding working memory components); which implies that the amplitudes of the N2 and P3 in the current study may be affected by task complexity, and this could potentially influence the internal consistency of the N2 and P3. Therefore, for future research it is important to examine the internal consistency of the N2 and P3 in three ways: (a) in a Go/No-Go task with lower complexity levels of the No-Go stimuli (e.g., a single Go and No-Go stimuli), see [15],[31]; (b) other cognitive control tasks eliciting the N2 (e.g., stop-signal task); and/or (c) a context-specific N2 and P3, e.g., [25].

Based on the present findings, we recommend including at least 20 and 14 trials when measuring the N2 and P3 in a Go/No-Go task, respectively. Further, we recommend that at least 8 trails are required to measure the ERN and Pe in an Eriksen Flanker task.

The current study was set up to examine the internal consistency of brain activity related to error processing and inhibitory control. In line with previous findings, we have similar advice for the N2/P3 and ERN/Pe [1],[8],[9],[11],[14],[22],[23]. However, replication is needed to uncover the internal consistency of especially the N2 for similar as well as different behavioral tasks to confirm our conclusions and generalize the findings to other tasks (e.g., stop-signal task). Lastly, we employed a number of commonly employed statistical approaches to determine the internal consistency of the N2, P3, ERN and Pe. Future research may further examine this issue using more sophisticated statistical methods (e.g., simulation based methods).
